# CRISPR‐mediated mutation of cytokinin signaling genes (*SlHP2* and *SlHP3*) in tomato: Morphological, physiological, and molecular characterization

**DOI:** 10.1002/tpg2.20542

**Published:** 2025-01-08

**Authors:** Abdullah Aydin, Bayram Ali Yerlikaya, Seher Yerlikaya, Nisa Nur Yilmaz, Musa Kavas

**Affiliations:** ^1^ Department of Agricultural Biotechnology, Faculty of Agriculture Ondokuz Mayis University Samsun Turkey

## Abstract

Synergistic and antagonistic relationships between cytokinins and other plant growth regulators are important in response to changing environmental conditions. Our study aimed to determine the functions of *SlHP2* and *SlHP3*, two members of cytokinin signaling in tomato, in drought stress response using CRISPR/Cas9‐mediated mutagenesis. Ten distinct genome‐edited lines were generated via *Agrobacterium tumefaciens*‐mediated gene transfer and confirmed through Sanger sequencing. Stress experiments were conducted with two of these lines (*slhp2,3‐10* and *slhp2,3‐11*), which harbored homozygous mutations in both genes. The responses of two lines carrying homozygous mutations in both genes under polyethylene glycol (PEG)‐induced stress were examined using morphological, physiological, biochemical, and molecular methods. The genome‐edited lines demonstrated enhanced water retention, reduced stomatal density, and less oxidative damage compared to the wild‐type plants under PEG‐induced stress. Moreover, the *slhp2,3* double mutant plants exhibited improved root growth, showcasing their superior drought tolerance over wild‐type plants by accessing deeper water sources and maintaining hydration in water‐limited environments. To investigate the involvement of cytokinin signaling regulators and genes associated with stomatal formation and differentiation, the expression of genes (*Speechless* [*SPCH*], *FAMA*, *MUTE*, *TMM*, *HB25*, *HB31*, *RR6*, *RR7*, and *Solyc02g080860*) was assessed. The results revealed that all regulators were downregulated, with *SPCH*, *TMM*, *RR7*, and *RR6* showing significant reductions under PEG‐induced stress. These results emphasize the promise of utilizing CRISPR/Cas9 to target cytokinin signaling pathways, enhancing drought tolerance in tomatoes through improvements in water retention and root growth, along with a reduction in stomatal density and malondialdehyde content.

## Core Ideas

- *SlHP2* and *SlHP3* genes negatively regulate root development.
- *SlHP2* and *SlHP3* genes negatively regulate abscisic acid signaling.
- These genes foster development and growth by favorably regulating the production of stomata and chlorophyll.

**Abbreviations**
AHK: Arabidopsis histidine kinase
AHP: Arabidopsis histidine‐containing phosphotransfer protein
APHP: Arabidopsis pseudo histidine‐containing phosphotransfer protein
ARR: Arabidopsis response regulator
CRE: cytokinin receptor
CRF: cytokinin response factor
gRNA: guide RNA
HP: histidine‐containing phosphotransfer protein
MDA: malondialdehyde
PEG: polyethylene glycol
PPI: protein‐protein interaction
RR: response regulator
RWC: relative water content
SPAD: Soil Plant Analysis Development
SPCH: Speechless
TCS: two‐component system
WUE: water use efficiency

## 1 INTRODUCTION

Cytokinins, which are N6‐substituted adenine derivatives, are essential regulators of diverse plant growth and developmental processes, including cell division (Sosnowski et al., 2023), shoot initiation (Cheng et al., 2013), vascular tissue development (Kieber & Schaller, 2010), leaf senescence (Kieber & Schaller, 2010), deetiolation (Chory et al., 1994), and chloroplast differentiation (O'Brien & Benková, 2013; Pospíšilová et al., 2000; Zwack & Rashotte, 2015). The signaling mechanism of cytokinins is proposed to follow a multistep phosphorelay model, akin to the two‐component systems (TCSs) observed in bacteria, allowing plants to sense and respond to environmental signals through sequential phosphorylation events (Stock et al., 2000; West & Stock, 2001). In a basic TCS, a His sensor kinase detects an external signal and autophosphorylates on a specific histidine residue. This phosphoryl group is subsequently transferred to an aspartate residue within the receiver domain of a response regulator (RR), modulating its activity to control downstream responses. In more complex phosphorelay pathways, additional His‐ and Asp‐containing modules facilitate the transfer of the phosphoryl group through a series of His‐Asp‐His‐Asp exchanges (Perraud et al., 1999). Following the autophosphorylation event, the receptor kinase transfers the phosphoryl group to a histidine‐containing phosphotransfer protein (HPt), which then relays the phosphate to nuclear‐localized RRs. Type‐B RRs act as transcription factors, driving the expression of genes involved in cytokinin‐mediated growth and developmental processes, while type‐A RRs function within a feedback loop to fine‐tune the signaling pathway (To & Kieber, 2008). In *Arabidopsis thaliana*, cytokinin receptors (CREs) such as CYTOKININ RESPONSE1 (CRE1), also known as WOODENLEG or ARABIDOPSIS HISTIDINE KINASE4 (AHK4), and their homologs AHK2 and AHK3 resemble bacterial hybrid histidine kinases, integrating ligand‐binding, His kinase, and receiver domains within a single protein structure (Inoue et al., 2001; Yamada et al., 2001). He et al. (2016) identified a total of 65 TCS genes in tomato. Among the 65 genes identified, 20 are histidine kinases (HKs), six are histidine‐containing phosphotransfer proteins (HPs), and 39 are RRs.

CREs play a pivotal role in mediating cytokinin responses in plants. Mutations that result in the loss of function of the CRE CYTOKININ RESPONSE1 (CRE1) lead to diminished sensitivity to cytokinins (Franco‐Zorrilla et al., 2002; Inoue et al., 2001). In plants with double mutations of CREs, the response to cytokinins is notably reduced, and in triple mutants, where all three CREs are disrupted, the plants become almost entirely insensitive to cytokinins. These mutants exhibit phenotypic abnormalities, including small shoots, a shortened primary root, and significantly reduced or absent seed production (Higuchi et al., 2004; Nishimura et al., 2004; Riefler et al., 2006). Research has demonstrated that cytokinins bind to the CHASE domain of Arabidopsis histidine kinases (AHKs), which activates their His kinase activity in yeast and bacterial systems, underscoring the importance of cytokinin binding in the functionality of these receptors (Inoue et al., 2001; Romanov et al., 2005; Spíchal et al., 2004; Yamada et al., 2001).

Arabidopsis response regulators (ARRs) are categorized into two main classes: type‐A and type‐B. Type‐B ARRs are characterized by a receiver domain and an extended C‐terminal region, which includes both a DNA‐binding domain and a transcription activation domain (Lohrmann et al., 1999; Sakai et al., 2000). A subset of type‐B ARRs has been identified as positive regulators of cytokinin responses. These type‐B ARRs directly activate the expression of cytokinin‐inducible type‐A ARRs, positioning them as key upstream activators in the cytokinin signaling pathway (Mason et al., 2005; Sakai et al., 2000). Type‐A ARRs are rapidly upregulated at the transcriptional level in response to cytokinin and are characterized by a receiver domain coupled with short C‐terminal extensions (Brandstatter & Kieber, 1998; Kiba et al., 1999; Taniguchi et al., 1998). Unlike type‐B ARRs, type‐A ARRs function as negative regulators of cytokinin signaling. Loss‐of‐function mutations in type‐A ARRs lead to heightened sensitivity to cytokinin (To et al., 2004). A total of 31 RR genes have been identified within the tomato genome (Y. Liu, Liu, et al., 2023). Genetic studies have revealed that, beyond their function in cytokinin signaling, certain type‐A ARRs also play crucial roles in regulating abiotic stress responses, circadian rhythms, and managing shoot apical meristem activity (Leibfried et al., 2005; Y. Liu, Liu, et al., 2023; Salome et al., 2006).

Another key element in cytokinin signaling involves the *CYTOKININ RESPONSE FACTOR* (*CRF*) genes, which encode transcription factors essential for the pathway (Hallmark & Rashotte, 2019; Rashotte et al., 2006). Similar to type‐A ARRs, *CRF* genes are transcriptionally upregulated in response to cytokinin through a mechanism dependent on type‐B ARRs. Cytokinin also affects the subcellular localization of CRFs, causing them to accumulate in the nucleus. This nuclear localization is mediated by AHKs and Arabidopsis histidine‐containing phosphotransfer proteins (AHPs), but interestingly, it does not require type‐B ARRs. Insertion mutants of CRF genes show a diminished induction of cytokinin‐responsive genes that overlap with those regulated by type‐B ARRs. However, despite this overlap, mutations in CRF genes do not significantly impair cytokinin responses in various cytokinin sensitivity assays (Rashotte et al., 2006).

The AHPs represent a family of six related proteins, with AHP1 through AHP5 containing the conserved amino acids necessary for their function as histidine phosphotransfer proteins (HPts). In contrast, APHP1 (where APHP is Arabidopsis pseudo histidine‐containing phosphotransfer protein), also known as AHP6, is considered a pseudo‐AHP due to its lack of the conserved histidine residue required for phosphorylation, rendering it inactive in standard phosphotransfer reactions (Mähönen et al., 2006; Suzuki et al., 2000). Several studies have demonstrated that AHPs are integral to cytokinin signaling, functioning as intermediaries that mediate phosphotransfer between CREs and both type‐A and type‐B RRs. In vitro and heterologous complementation experiments have confirmed that AHPs can act as phosphorelay intermediates (Miyata et al., 1998; Suzuki et al., 1998, 2000). They can be phosphorylated by AHKs and, in turn, can phosphorylate both type‐A and type‐B ARRs, indicating their role in cytokinin‐responsive pathways. Additionally, AHP1, AHP2, and AHP4 have been observed to accumulate in the nucleus in response to cytokinin, further supporting their involvement in cytokinin signaling within plant cells (Hwang & Sheen, 2001; Yamada et al., 2004). The most direct evidence of HPts mediating cytokinin signaling in plants was provided by experiments with cultured periwinkle cells, where the cytokinin‐induced expression of an RR was reduced upon silencing a His phosphotransfer protein via RNA interference (Papon et al., 2004). Moreover, overexpression of AHP2 has been associated with a slight increase in cytokinin sensitivity in root elongation assays, reinforcing the role of AHPs in cytokinin signaling (Suzuki et al., 2002). On the other hand, recent genetic analysis has revealed that APHP1/AHP6 acts as a negative regulator of the cytokinin response pathway (Mähönen et al., 2006), likely through a dominant negative mechanism.

**Core Ideas**

- *SlHP2* and *SlHP3* genes negatively regulate root development.
- *SlHP2* and *SlHP3* genes negatively regulate abscisic acid signaling.
- These genes foster development and growth by favorably regulating the production of stomata and chlorophyll.

Abiotic stresses, such as drought, severely impact the growth and productivity of key agricultural crops, leading to substantial yield reductions that can contribute to food shortages and jeopardize the sustainability of agriculture (Aroca, 2012; Farooq et al., 2009; Seki et al., 2004; Wahid et al., 2009). Plants have limited capabilities to adapt to drought, involving rapid morphological responses (like root development, stomatal decrease, and stomatal closure) and phenotypic changes (such as decreased malondialdehyde [MDA] content, increased reactive oxygen species scavangers like proline and antioxidaants, enzymatic activities, etc.) (Chaves et al., 2003; Farooq et al., 2009; Osakabe et al., 2014), often mediated by phytohormones. Among the various phytohormones, cytokinin plays a pivotal role as a central mediator in plant responses to abiotic stress, orchestrating a range of functions that allow plants to adapt to diverse stress conditions (Cortleven et al., 2019; W. Li, Herrera‐Estrella et al., 2019). Cytokinins have been shown to exert a dual role in plant drought tolerance. Initially, cytokinins were reported to enhance drought tolerance by increasing endogenous cytokinin levels in transgenic plants during the early stages of drought (Rivero et al., 2007). However, more recent studies involving cytokinin‐deficient and cytokinin‐signaling mutants have revealed that cytokinins can also negatively influence plant responses to drought (Cortleven et al., 2019; W. Li et al., 2016; Ramireddy et al., 2018). This suggests that cytokinins have multifaceted functions, acting both as promoters and inhibitors of drought tolerance in plants (Prerostova et al., 2018). Understanding cytokinin signaling pathways in economically significant plants such as tomato could provide opportunities for biotechnological interventions aimed at improving drought resistance. Tomato, with its widespread cultivation and biological characteristics, serves as an important model organism for research in this area (Y. Liu et al., 2020; M. Liu, Zhao, et al., 2023). In tomato, two of the six different HP coding genes were identified as pseudogenes (He et al., 2016). Meaning that there are four different HPs functionally involved in cytokinin signaling. The expression of *SlHP2* and *SlHP3* increased in leaves under drought stress, whereas under normal conditions, *SlHP2* was expressed in root, leaf, and flower tissues, while *SlHP3* was expressed in root, flower, and fruit tissues. Therefore, SlHP2 and SlHP3, positive regulators in cytokinin signaling, were chosen to target by CRISPR/Cas9 to respond to osmotic stress.

Polyethylene glycol (PEG)‐6000 is commonly used for simulating drought and osmotic stress in plants because it creates an osmotic potential that reduces water uptake without being toxic. Its high molecular weight prevents water absorption by plant tissues, mimicking drought conditions effectively. PEG‐6000 has been shown to influence key physiological processes like seed germination, shoot/root elongation, photosynthesis, and stomatal development, making it ideal for drought and osmotic stress tolerance screening in plants (Licaj et al., 2024; Pavli, 2020; Peršić et al., 2022).

Stomatal development results from the division of distinct cell types and is primarily controlled by bHLH transcription factor family proteins, including Speechless (SPCH), Mute, and FAMA. (Shimazaki et al., 2007). Stomatal development begins with the differentiation of mother meristemoid cells into meristemoid cells, facilitated by SPCH. Afterward, MUTE terminates asymmetric division and induces the differentiation of meristemoid cells into guard mother cells. The differentiation of guard mother cells into mature guard cells is controlled by the FAMA transcription factor (Negi et al., 2014). Farber et al. (2016) found that decreased endogenous cytokinin levels disrupted stomatal development. The characteristic gene expressions of *SlHP2* (*Solyc01g080540*) and *SlHP3* (*Solyc06g084410*) genes and the fact that their gene expressions were induced under drought indicated the need to investigate the functions of these genes. This study represents the first examination in the literature of the roles of SlHP2 and SlHP3, two members of cytokinin signaling, through CRISPR/Cas9‐mediated mutagenesis in tomato. We investigated the morphological, biochemical, and molecular responses of the slhp2,3 double mutant plants to PEG‐induced osmotic stress.

## 2 MATERIALS AND METHODS

### 2.1 Guide RNA design and CRISPR/Cas9 vector construction

To design guide RNAs (gRNAs), we retrieved *SlHP2* (*Solyc01g080540*) and *SlHP3* (*Solyc06g084410*) gene sequences from the NCBI database. In order to identify the appropriate potential gRNAs, Benchling (https://benchling.com/) and CRISPR‐P version 2.0 software (H. Liu et al., 2017) were performed. Bencling (Gooden et al., 2021) is a robust tool for general CRISPR gRNA design, offering a user‐friendly interface and comprehensive data management features. However, discrepancies may arise between predicted and actual outcomes, and off‐target effects may not always be fully anticipated. On the other hand, CRISPR‐P (Bradford & Perrin, 2019) is specifically optimized for plant genome editing, providing more reliable on‐target and off‐target predictions, but variability in efficiency across plant species and data gaps for certain species necessitate experimental validation. Our selection of four targets (with two gRNAs designated for each) was guided by factors such as cytosine and guanine content, gene location, off‐target potential, the presence of A or T bases at the 17th nucleotide, and the secondary structure of gRNAs (Figure S1a–d). The regions targeted by the selected gRNAs are presented in Figure S1e.

The pHSE401 expression vector (Addgene plasmid # 62201) was employed in the Golden Gate Cloning method for expressing gRNAs and Cas9 in plants, as extensively detailed by Xing et al. (2014). To assemble four gRNAs into pHSE401, we used 10 primers (refer to Table S1) for three different PCR amplifications (DT1, DT2, and DT3T4). This amplification process was conducted using Q5 High‐Fidelity DNA Polymerase (NEB), following the manufacturer's guidelines. After purifying the PCR products from agarose gel, we assembled them into pHSE401 using the Golden Gate cloning method, employing BsaI and T4 DNA Ligase (NEB) per the manufacturer's instructions.

The binary vector, named pHSE401‐*SlHP2*,*3* (Figure S2a), which contains gRNA3, gRNA25, gRNA29, and gRNA78, was subsequently used to transform Dh5α competent *Escherichia coli*. Plasmids were isolated using the GeneJET Plasmid Miniprep Kit from Thermo Scientific. Positive clones were verified through PCR using gene‐specific primers, and Sanger sequencing confirmed the presence of positive clones. Finally, the constructed plasmid, pHSE401‐*SlHP2*,*3*, was introduced into the *Agrobacterium tumefaciens* strain *GV3101* using electroporation.

### 2.2 *Agrobacterium*‐mediated transformation

*Agrobacterium*‐mediated transformation and tissue culture steps were conducted according to the methods described by Secgin et al. (2022) and Gökdemir et al. (2022). Tomato seeds of the Crocker cultivar (indeterminate variety) were sourced from Syngenta. The transformation of tomato plants (*Solanum lycopersicum* cv. ‘Crocker’) was carried out using the *A*. *tumefaciens*‐mediated method. In brief, cotyledon explants aged between 8 and 10 days were immersed in an *Agrobacterium* suspension culture and subsequently transferred to a co‐culture medium containing 0.5 mg/L indole‐3‐acetic acid (IAA), 3 mg/L benzyl adenine (BA), 100 mg/L acetosyringone, and 100 mg/L ascorbic acid. They were then incubated in darkness at 25  ±  2°C for 2 days. Following the co‐culture, the explants were transferred to a Murashige and Skoog (MS) medium supplemented with 3 mg/L BA, 0.5 mg/L IAA, 10 mg/L hygromycin, 100 mg/L ascorbic acid, 160 mg/L timentin, and 125 mg/L cefotaxime, and incubated at 25°C to stimulate the regeneration. After 4 weeks, the regenerated shoots measuring 2–4 cm in length were transferred to a rooting medium composed of MS, 0.5 mg/L IAA, 10 mg/L hygromycin, and 160 mg/l timentin. Healthy plants were ultimately transplanted into pots containing a soil and peat mixture in a 2:1 ratio. All tomato lines were cultivated in a standard greenhouse with a 16‐h day length and temperature maintained between 24 and 26°C during the day and 18°C at night. Plants from each line were grown in 30‐L pots filled with coarse potting compost (Klasmann Potgrond H) and received standard Hoagland's solution irrigation treatment. Tomato fruits were harvested at commercial maturity and used for subsequent analyses.

### 2.3 Verification of CRISPR/Cas9‐Induced mutations

Genomic DNA was isolated from the leaves of wild‐type (WT) plants and transformants using the GeneJET Plant Genomic DNA Purification Kit (K0791). To confirm the presence of transgenes in T_0_ plants, PCR was performed utilizing gene‐specific primers for *HPTII* (refer to Table S2). Figure S2b provides an agarose gel picture of the PCR findings using particular primers for the *HPTII* gene. The PCR protocol to amplify *HPTII* encoding gene consisted of an initial denaturation at 94°C for 3 min, followed by 30 cycles of denaturation at 94°C for 15 s, annealing at 65°C for 30 s, and extension at 68°C for 30 s. A final extension was performed at 68°C for 5 min. To identify CRISPR/Cas9‐induced mutations, specific PCR primers were used to amplify genomic regions spanning the targeted *SlHP2* and *SlHP3* regions (Table S2), followed by analysis. The amplified PCR products were subjected to Sanger sequencing to detect potential mutations. Comparative analysis was carried out by aligning the obtained sequences with the WT reference sequence of the *SlHP2* and *SlHP3* genes using BLASTN from NCBI. The Synthego ICE CRISPR Analysis Tool (Conant et al., 2022) and DSDecodeM (W. Liu et al., 2015) were used to calculate the mutation rates in transgenic lines.

### 2.4 PEG‐6000 treatment

To induce osmotic stress, the protocol developed by R. Li, Liu et al. (2019) using PEG6000 was applied to tomato. The experiment was conducted under greenhouse conditions in Samsun, Turkey, at a temperature of 27 °C ± 1. *Slhp2,3‐10* (mutant line 10), *slhp2,3‐11* (mutant line 11), and WT (Crocker) tomato seeds were sown at a depth of 3 cm in pots measuring 13 cm × 13 cm × 13 cm, containing a mixture of peat and vermiculite in a 2:1 ratio. The seedlings were irrigated with 1/2 Hoagland solution until they developed five mature leaves. The height measurements of the plants that had developed five mature leaves were conducted using a tape measure. Subsequently, the mutant and WT plants were divided into two groups: well‐watering and PEG‐treating. The plants designated for well‐watering were irrigated with 1/2 Hoagland solution for 7 days, while the PEG group was watered with 1/2 Hoagland solution containing 20% PEG_6000_. The irrigation process was carried out until the soil in the pots reached field capacity.

### 2.5 Data collection and processing

To evaluate the roles of the *SlHP2* and *SlHP3* genes in transgenic tomato plants under water‐deficient conditions, a variety of morphological and biochemical parameters were assessed. At the end of the 7‐day period, the plants were photographed, and their height measurements were taken using a tape measure. Samples for RNA isolation, MDA, proline, relative water content (RWC), electrolyte leakage, and water‐loss experiments were taken from the third most developed leaves. The samples for RNA, MDA, and proline analyses were frozen using liquid nitrogen. After being separated from the soil with the aid of water and soap, the plant roots were left in water for 24 h. At the end of the 24‐h period, the weights of the plant roots were measured. The plant roots were scanned using an LA2400 scanner and then analyzed with the WinRHizo analysis software. The root length measurements represent the combined total of primary and lateral root lengths for each replicate, recorded in centimeters.

Lipid peroxidation‐induced membrane damage was assessed by quantifying the MDA content, following the procedure outlined by Hodges et al. (1999) and Kavas et al. (2020). To analyze proline levels, 0.25 g of leaf tissue from both the control and treatment groups was homogenized using liquid nitrogen. The resulting tissue powders were suspended in 1 mL of 3% sulphosalicylic acid for proline content measurement, following the method described by Bates et al. (1973).

RWC measurement was conducted according to M. M. Jones and Turner (1978) protocol. Leaf discs with a 2‐cm radius were taken, and fresh weight was measured. The weighed leaf discs were placed in 50‐mL Falcon tubes containing 15 mL of deionized water for 24 h to obtain their turgid weight. Subsequently, they were dried at 70°C for 48 h to measure dry weight. The formula used for RWC (%) is [(Fresh Weight − Dry Weight)/(Turgid Weight − Dry Weight)] × 100.

For water‐loss measurements, third leaves were detached. The fresh weights of the detached leaves were measured and then kept at 25°C under a light source, with the adaxial parts of the leaves facing upwards. For 4 h, leaf weights were measured every hour. The water loss was calculated using these data and expressed as percentage. The formula used for water loss calculation: [(Fresh Weight − first or second or third or fourth weight)/Fresh Weight)] × 100.

Electrolyte leakage was calculated using the conductivity method (Lafuente et al., 1991). Leaf samples of 1 cm^2^ taken from the same region were placed in Falcon tubes containing 15 mL of deionized water and left at room temperature for 24 h. Subsequently, the conductivity of the solution in the Falcon tubes was measured using a conductivity meter (C1). The tubes were then incubated at 95°C for 15 min, and the conductivity was measured again (C2). The formula used to calculate electrolyte leakage (%) is (C1/C2) × 100.

Imaging was conducted to determine the total leaf area of the plants. The leaf area in the images was calculated using the ImageJ software (Schindelin et al., 2012).

Leaves taken from the same regions were transferred to the fixative solution under a vacuum, and the samples were later lyophilized for imaging using a scanning electron microscope (JSM‐7001F). The width, length, and aperture of the stomatal structures were analyzed using the ImageJ software (Schindelin et al., 2012).

### 2.6 Unveiling genetic interactions: Co‐expression network analysis and ortholog identification in Arabidopsis

Bidirectional BLAST alignments were performed to analyze protein sequences in *S*. *lycopersicum* and *A*. *thaliana*. Our criteria for orthologous search were as follows: the top hit in aminoacid blast alignment was chosen as the orthologous pairs. Table S3 provides the outcomes of the orthologous search.

The ATTED‐II NetworkDrawer (Obayashi et al., 2007) was utilized to generate the co‐expression network of cytokinin signaling and stomata differentiation genes. In this analysis, the co‐expression option was configured to “add a few genes,” while the PPI (protein–protein interaction) option was set to “Draw PPIs.”

### 2.7 Real‐time qPCR analysis

Total RNA extraction was carried out from leaf tissues employing the GeneJET Plant RNA Purification Kit (K0801). Subsequently, 1 µg of RNA was utilized for the synthesis of first‐strand cDNA using the Biorad iScript cDNA Synthesis Kit (1708891), following the manufacturer's instructions. For the quantitative reverse transcription (qRT)‐PCR, a Bio‐Rad CFX 96 Real‐Time Detection System was employed, along with Promega GoTaq qPCR Master Mix (A6001). The thermal cycling protocol consisted of an initial denaturation step at 95°C for 2 min, followed by 40 cycles of denaturation at 95°C for 15 s and annealing/extension at 60°C for 1 min. To ensure robustness and reliability, three biological replicates, as well as two technical replicates, were performed for both mutant T_0_ lines and WT plants. Additionally, negative controls with no template were included in each run to identify and mitigate potential contamination. The reference genes used in this study were *SlActin* (*Solyc03g078400*) and *SlUbiquitin* (*Solyc01g056940*), and relative expression changes were determined utilizing the 2^−ΔΔCt^ method described by Livak and Schmittgen (2001). Primer sequences utilized in this analysis are provided in Table S4. Gene expressions calculated according to qRT‐PCR results are given as mutant/WT in the same conditions, while only the expressions of *9‐cis‐epoxycarotenoid dioxygenase* (*NCED*) and *RD22* genes are given as PEG/Control.

### 2.8 Statistical analysis

In this study, statistical analyses were carried out to assess variations among different groups and all experiments were conducted as three biological replicates. For comparisons involving multiple groups, a two‐way analysis of variance was used to detect any statistically significant differences. If significant differences were observed, post hoc analyses were performed using the Tukey test to pinpoint specific group differences. For comparisons between two groups, an independent samples *t*‐test was employed to determine if there was a statistically significant difference between their means. All statistical procedures were conducted using IBM SPSS Statistics software, with a significance threshold set at *p* < 0.05. Data were presented as mean ± standard deviation (SD).

## 3 RESULTS

### 3.1 The CRISPR/Cas9‐engineered mutations in *SlHP2* and *SlHP3* resulted in double homozygous mutations

**FIGURE 1:**
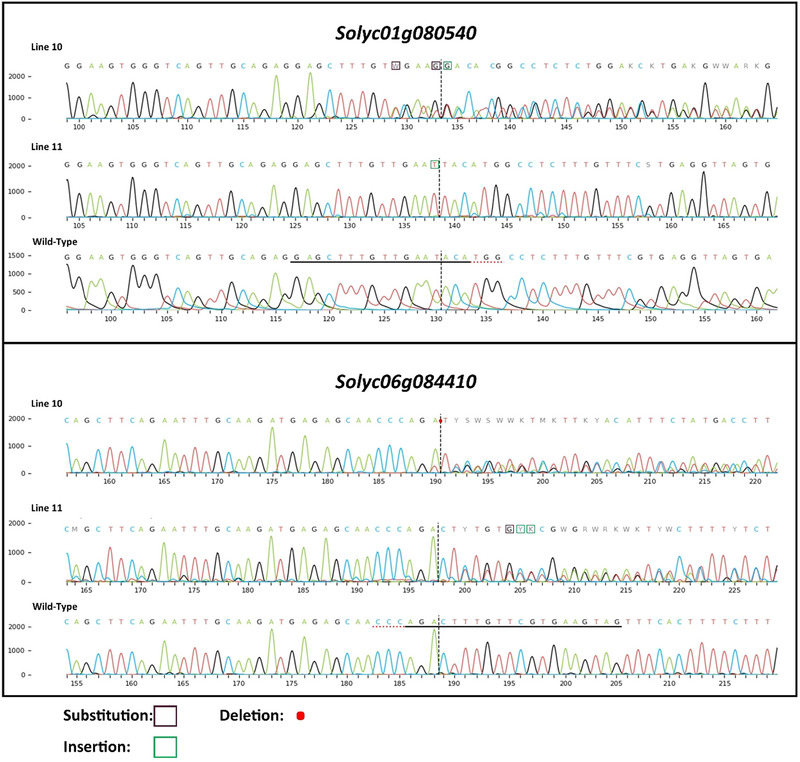
The pattern of mutations in the *SlHP2* and *SlHP3* locus generated by the CRISPR/Cas9 genome editing system. The analysis conducted using the ICE‐Synthego tool. The dashed red lines represent the protospacer adjacent motif sequence, while the solid black line indicates the target region, and the dashed vertical line represents the predicted cleavage site. The shapes used for the representation of mutations depict black squares for base substitutions, green squares for base insertions, and red dots for base deletions. Sanger sequencing chromatograms of the edited regions show distinct peak patterns corresponding to different nucleotide variations induced by CRISPR editing. The ICE tool was used to analyze these variations, quantifying InDels, and identifying heterozygous mutations based on the observed peak shifts. Nucleotide ambiguities are represented using International Union of Pure and Applied Chemistry (IUPAC) codes: W (A or T), S (G or C), M (A or C), K (G or T), R (A or G), Y (C or T), B (C, G, or T), D (A, G, or T), H (A, C, or T), V (A, C, or G), and N (A, C, G, or T). These codes reflect heterozygosity and InDel events at specific positions within the targeted gene region.

Two gRNAs, namely, gRNA3 and gRNA25, were designed to target the *SlHP2* gene, while another pair of gRNAs, gRNA29 and gRNA78, were selected to target the *SlHP3* gene, adhering to predetermined criteria. Subsequently, these gRNAs were incorporated into a plant expression vector designated as pHSE401 through the process of golden gate cloning (Figure S2a). To mutate *SlHP2* and *SlHP3* genes, a total of 150 8–10 days old cotyledon explants were transformed with *A*. *tumefaciens* GV3101 carrying pHSE401 plasmid harboring four gRNAs. Following our optimized regeneration protocol, regenerated plantlets were obtained. Ten potential genome‐edited transgenic lines were identified by PCR using hygromycin primers and genomic DNA isolated from putative transgenic plants transferred to soil (Figure S2b). Following the identification of transgenic plants, the genomic regions encoding *SlHP* genes were amplified using PCR to enable the detection of mutations. Subsequently, these PCR products were sequenced using the Sanger sequencing. Upon examination of the DNA sequencing results, 10 lines were analyzed and mutations were detected in both gene regions in the 10th and 11th lines using ICE‐Synthego Tool (Figure S3). The Sanger sequencing data obtained for the mutated regions revealed the presence of various peak patterns, indicating distinct nucleotide variations. These variations were quantified and categorized using specific nomenclature to assess the outcomes of the CRISPR‐induced alterations (Figure 1). The two lines where mutations were detected were utilized for subsequent experiments (Figure 1). The ICE (Inference of CRISPR Edits) program provides precise, experimental results for CRISPR mutation verification using Sanger sequencing, offering more reliable outcomes compared to predictive analyses. By quickly detecting mutations in the target region, ICE is a crucial tool in confirming the efficiency of CRISPR/Cas9 technology (Conant et al., 2022). Figure 1 displays the outcomes of the ICE‐Synthego mutation analysis tool only for 10th and 11th lines.

### 3.2 The *slhp2,3* mutant plants exhibited enhanced physiological and morphological adaptations to drought stress

**FIGURE 2:**
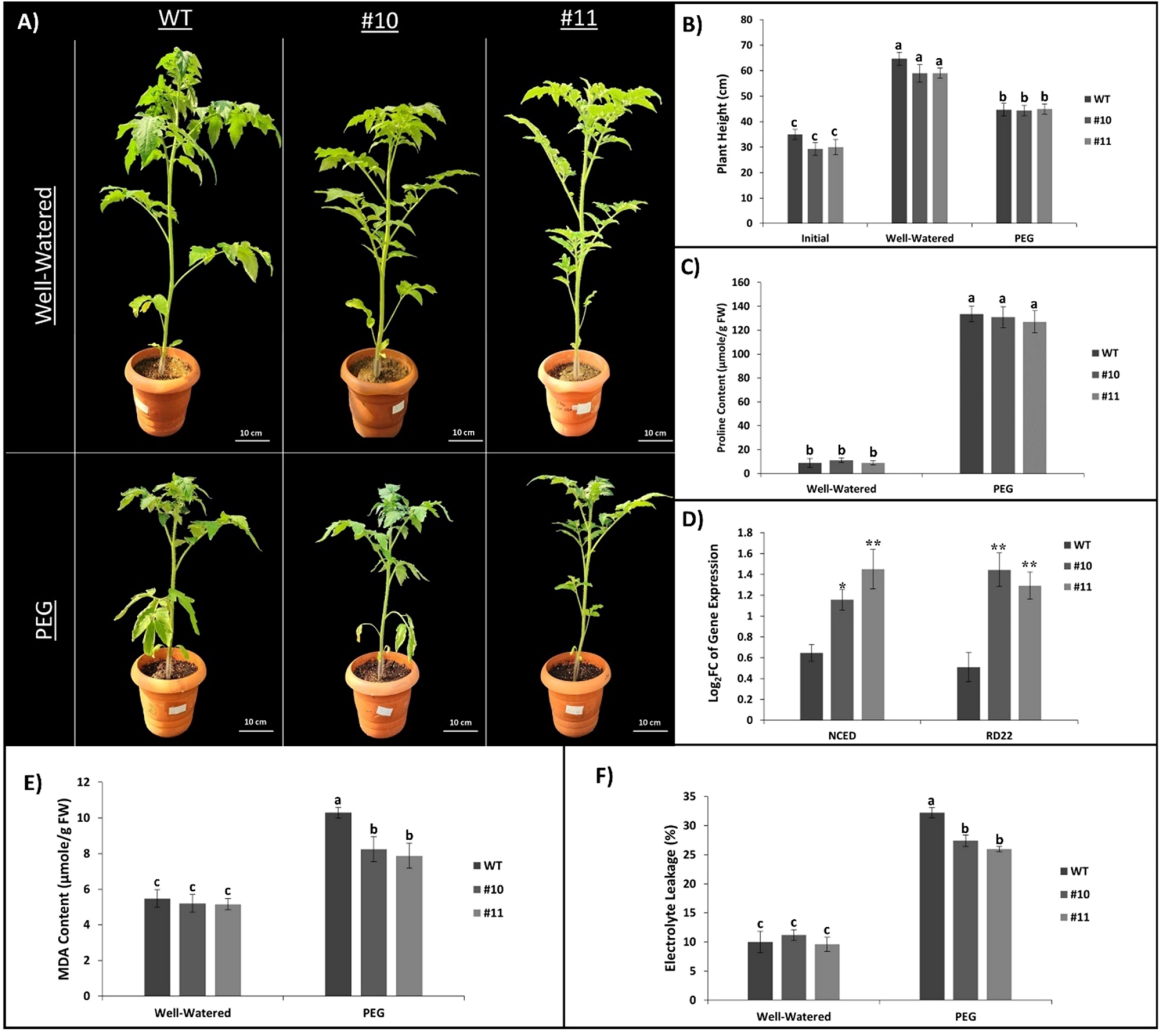
Physiological and morphological effects of polyethylene glycol (PEG)‐induced osmotic stress on mutant and wild‐type (WT) plants. (A) Mutant lines 10 and 11 were subjected to stress, along with WT plants. (B) The initial heights of WT and mutant lines at the start of the experiment and their heights following the treatments. (C) Proline contents of WT and mutant lines following stress and well‐watered conditions. (D) Expression of *NCED* and *RD22* marker genes in stressed plants presented as Log2FC compared to well‐watered plants. Asterisks indicate the significance of gene expression in mutant lines compared with the WT (*p < 0.05; **p < 0.01). (E) Assessment of the effects of abiotic stress on malondialdehyde (MDA) levels revealed different stress responses in transgenic and wild‐type lines. (F) Validation of membrane damage through the detection of electrolyte leakage. Black lines on (B)–(F) show SE ± values for three biological replicates. Different letters above the bars indicate statistically significant differences between groups (*p <* 0.05), as determined by ANOVA followed by Tukey's HSD test. Groups sharing the same letter are not significantly different.

The morphology of plants subjected to PEG‐induced stress was examined, and Figure 2A illustrates the plant morphologies. Prior to stress, when the WT, *slhp2,3‐10*, and *slhp2,3‐11* plants reached the five‐leaf stage, their average plant heights were 35, 29, and 30 cm, respectively. Following the treatment, the average heights for WT, *slhp2,3‐10*, and *slhp2,3‐11* under well‐watered conditions were 65, 59, and 59 cm, each, whereas the average heights for PEG‐treated plants were 45, 44, and 45 cm, respectively (Figure 2B). In terms of the osmoprotectant proline content (Figure 2C), no significant difference was observed under control and stress; however, under well‐watered conditions, proline contents were determined to be 9 µmole/gFW for WT, 10 µmole/gFW for *slhp2,3‐10*, and 6 µmole/gFW for *slhp2,3‐11*. Under PEG conditions, the proline contents were measured as 134 µmole/gFW, 131 µmole/gFW, and 127 µmole/gFW for WT, *slhp2,3‐10*, and *slhp2,3‐11*, respectively (Figure 2C). Significant differences were observed in the expression levels of the *NCED* and *RD22* genes, which are considered markers in response to drought stress (Figure 2D). These marker genes were significantly upregulated in the mutant lines. When examining the expression of the NCED gene presented as Log2FC, the gene expression levels were 0.65, 1.16, and 1.45 for WT, *slhp2,3‐10*, and *slhp2,3‐11* plants, respectively. For the *RD22* gene, the expression levels were calculated as 0.51, 1.45, and 1.29 for WT, *slhp2,3‐10*, and *slhp2,3‐11*, respectively (Figure 2D). In terms of MDA content (Figure 2E), no significant difference was observed under well‐watered conditions; however, under stress conditions, the mutant lines produced significantly less MDA compared to WT. Under stress conditions, WT plants produced an average of 10.3 nmole/g FW MDA, while the mutant lines produced 6.2 and 5.9 nmole/g FW, respectively (Figure 2E). When electrolyte leakage (%) was analyzed (Figure 2F), the results aligned with the MDA content findings. Under stress conditions, the mutant lines showed significantly lower leakage compared to the WT. Under stress conditions, the amount of electrolyte leakage from WT plant leaves were 32%, while it was found to be 27% and 26% in *slhp2,3‐10* and *slhp2,3‐11*, respectively (Figure 2F).

**FIGURE 3:**
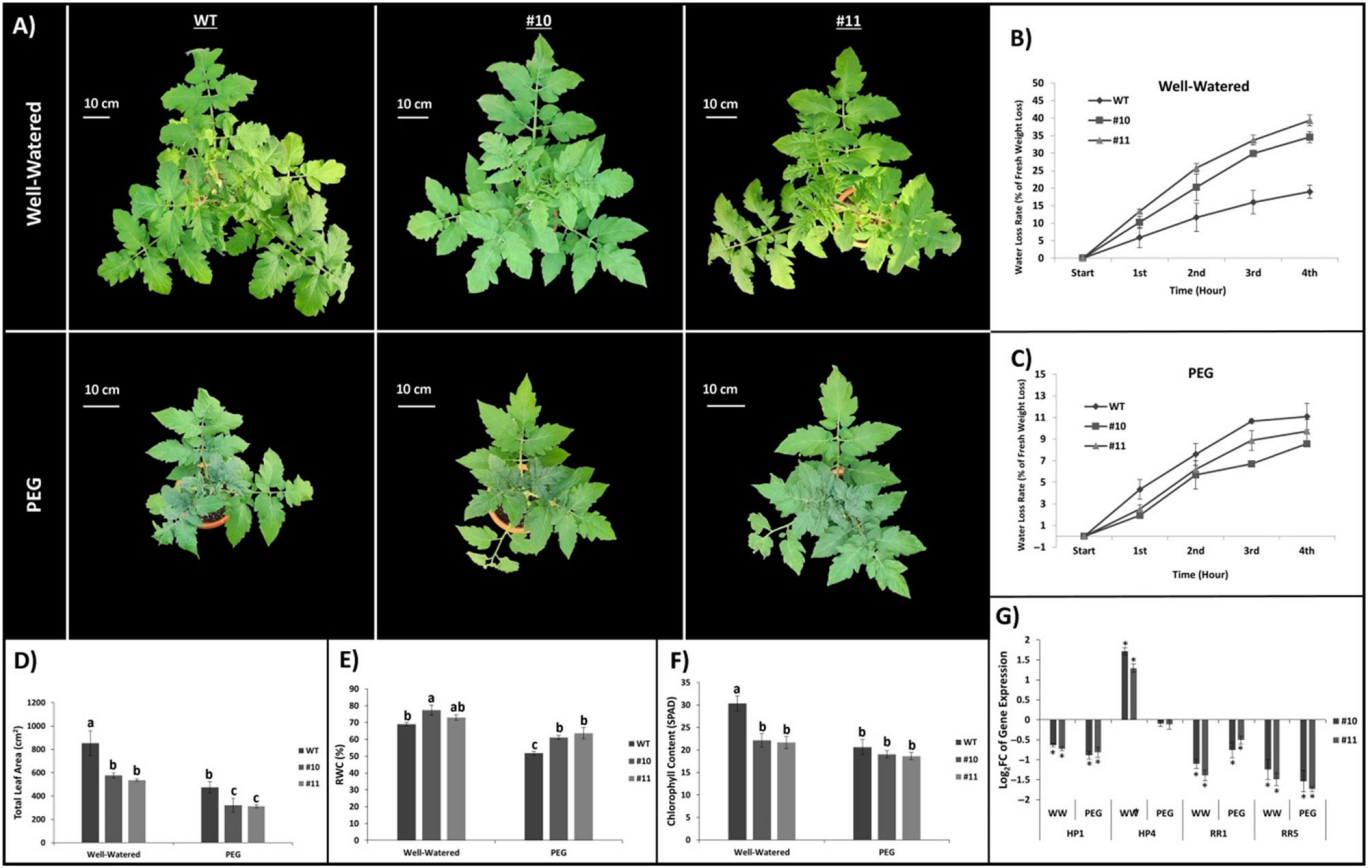
Comparison of wild‐type (WT) and mutant lines in terms of morphological and molecular parameters. (A) The aerial view of the plants at the end of the experiment. (B and C) The percentage of water loss in plants under well‐watered and polyethylene glycol (PEG)‐treated conditions. (D) The total leaf area produced by WT and mutant plants under well‐watered and PEG conditions. (E) Relative water content of fresh leaves. (F) SPAD measurement. (G) Expression of cytokinin signaling genes in mutants compared to WT under the same conditions. Asterisks indicate the significance of gene expression in mutant lines compared with the WT (*p < 0.01). Black lines on (B)–(F), and g show SE ± values for three biological replicates. Different letters above the bars indicate statistically significantdifferences between groups (*p* < 0.05), as determined by ANOVA followed by Tukey's HSD test. Groups sharing the same letter are not significantly different.

Following the completion of the treatment, when the leaf area of the plants was observed from above, a difference was noted between mutant plants and WT plants (Figure 3A). The water loss experiment quantified the rate of water loss in plant leaves. Figure 3B illustrates the percentage of initial weight lost by leaves under well‐watered conditions over a 4‐h period. Notably, mutant leaves exhibited significantly higher water loss compared to WT leaves. According to these results, while the WT plant leaf lost 18.97% of its weight after 4 h, *slhp2,3‐10* and *slhp2,3‐11* lost 34.58% and 39.31% of their weight, respectively (Figure 3B). The results were different in PEG‐irrigated conditions (Figure 3C). WT plant leaves exhibited a higher percentage of water loss, losing 11.1% of their weight. In contrast, *slhp2,3‐10* and *slhp2,3‐11* demonstrated improved water holding, with losses recorded at 8.55% and 9.7%, respectively (Figure 3C). Total leaf areas under both conditions are shown in Figure 3D. When the total leaf areas are examined, it is seen that WT plants have a larger leaf area compared to the mutants. The total leaf area for WT was 853 cm^2^ under well‐watered conditions and 475 cm^2^ under PEG conditions. However, for *slhp2,3‐10*, the leaf area was 576 cm^2^ and 321 cm^2^, and for *slhp2,3‐11*, it was calculated as 537 cm^2^ and 312 cm^2^, respectively. When RWC was assessed, mutant plants consistently exhibited significantly higher RWC compared to WT under both control and stress conditions. WT leaves had an RWC of 69% under well‐watered conditions and 52% under PEG conditions. In contrast, *slhp2,3‐10* showed RWC values of 77% and 61%, while *slhp2,3‐1* recorded 73% and 64%, respectively (Figure 3E). Soil Plant Analysis Development (SPAD) measurements revealed that the chlorophyll content in WT plant leaves was significantly higher compared to the mutant lines under well‐watered conditions. However, while a decrease in chlorophyll content was observed across all lines under PEG conditions, the differences were not statistically significant (Figure 3F). The expression levels of genes involved in cytokinin signaling were compared between mutant lines and WT plants (Figure 3G). While a general decrease in cytokinin signaling gene expression was observed in the mutant plants compared to the WT, it was notable that the *HP4* gene showed unexpectedly higher expression in the WT plants under well‐watered conditions.

### 3.3 *slhp2slhp3* double mutant tomato plants displayed increased root development under both control and drought stress conditions

**FIGURE 4:**
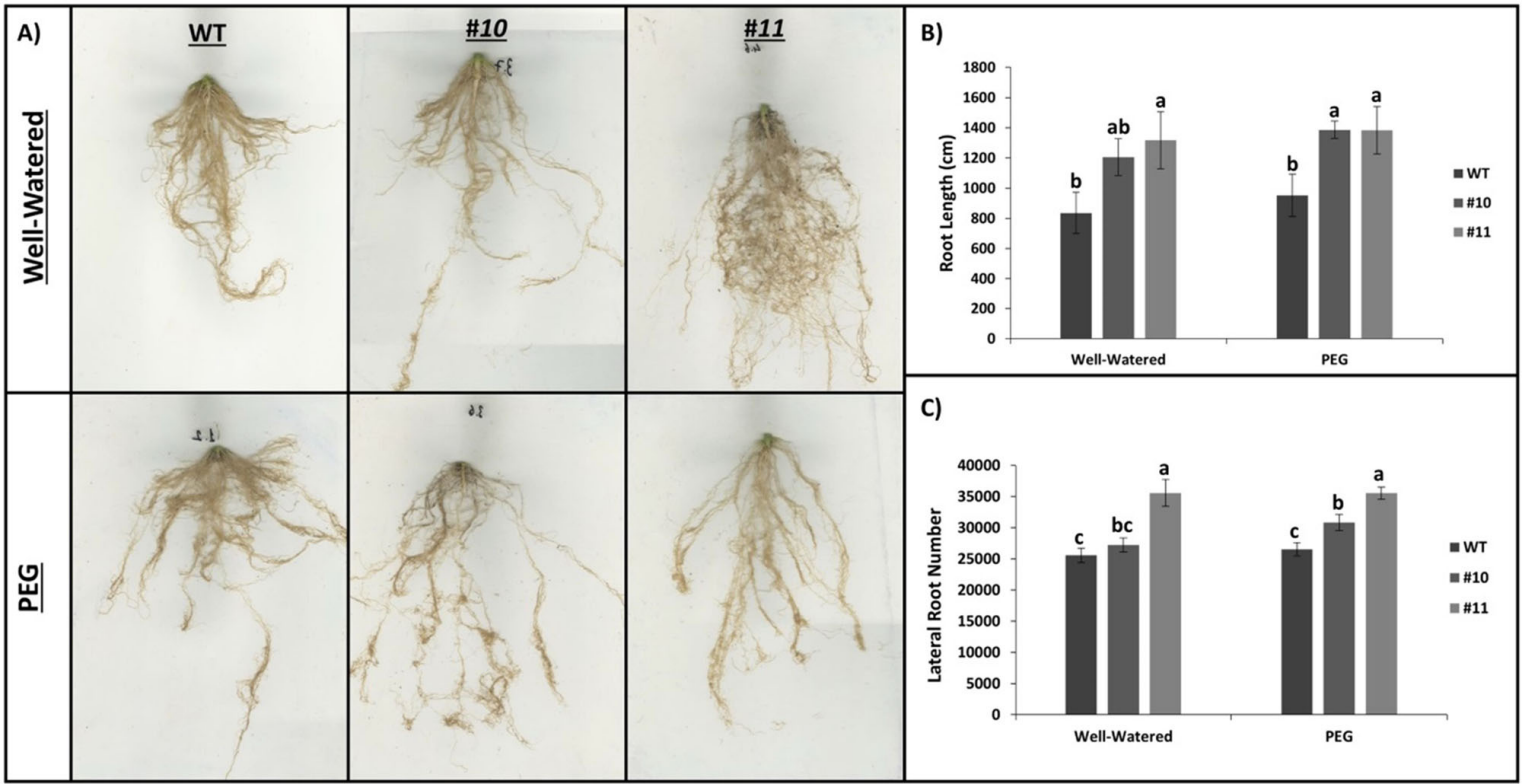
Root development of mutant lines and wild‐type (WT). (A) Displays of WT and mutant plant roots under drought and control conditions. (B) Main root length of WT and mutant plants under drought and control conditions. (C) Lateral root numbers of WT and mutant plants under drought and control conditions. Black lines on B and C show SE ± values for three biological replicates. Different letters above the bars indicate statistically significant differences between groups (*p* < 0.05), as determined by ANOVA followed by Tukey's HSD test. Groups sharing the same letter are not significantly different.

Root development, a key morphological trait linked to drought tolerance, was investigated (Figure 4). It has been noticed that mutant plants develop significantly increased primary roots compared to WT under both conditions (Figure 4A,B). WT plants have been observed to have root lengths of 835 and 952 cm under well‐watered and PEG conditions, respectively (Figure 4B). However, *slhp2,3‐10* developed primary roots of 1206 and 1387 cm, while *slhp2,3‐11* developed primary roots of 1317 and 1383 cm. In Figure 4C, the lines are compared in terms of the number of lateral roots. In terms of lateral roots, it has been observed that mutant plants have a significantly increased number of lateral roots compared to WT under both conditions. When comparing the mutant lines with each other, *slhp2,3‐11* is seen to develop higher number of lateral roots than *slhp2,3‐10* (Figure 4C).

**FIGURE 5:**
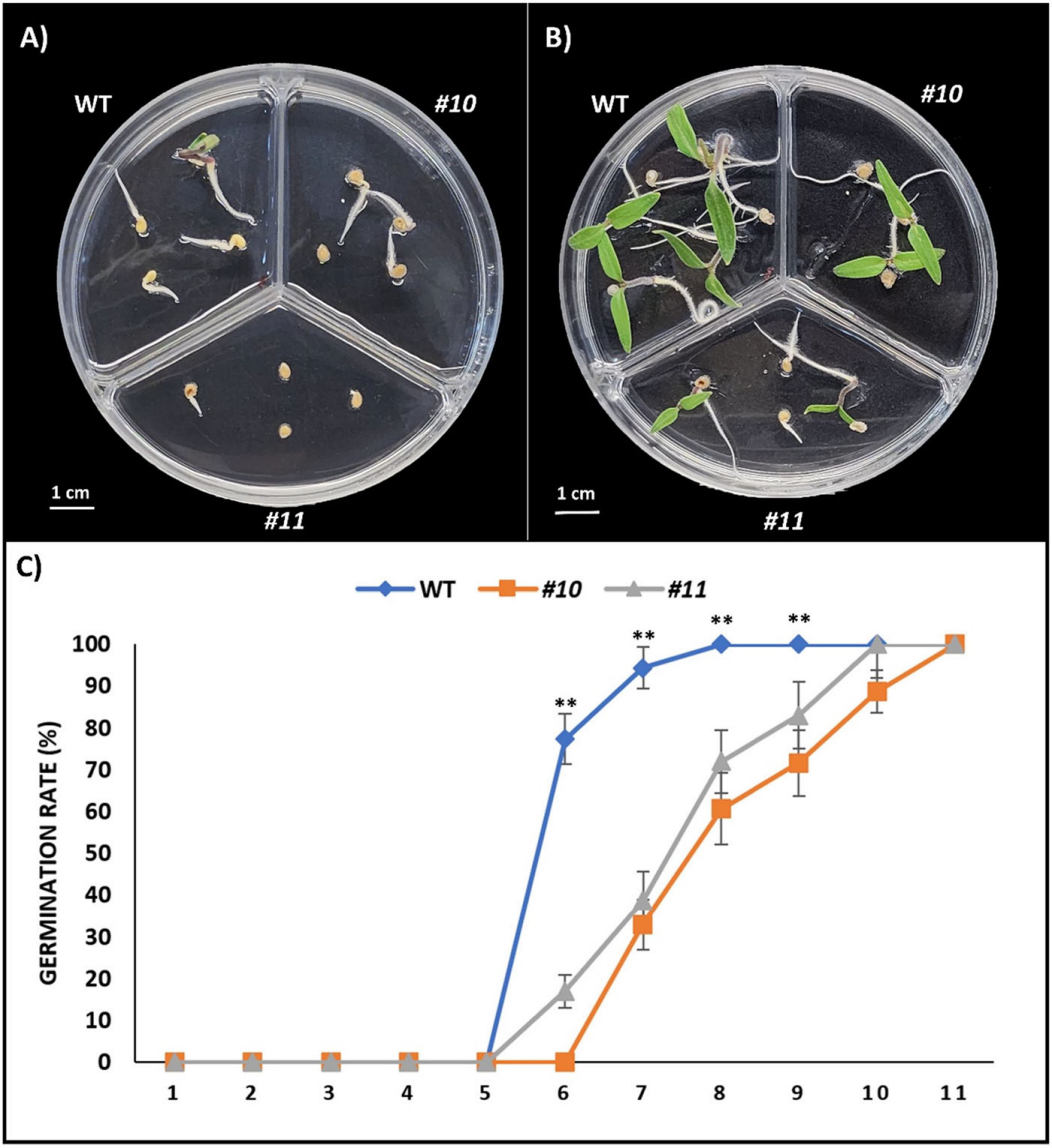
The germination rate of mutant lines and wild‐type (WT). (A) Displays of WT and mutant seeds 5 days after being planted in ½ MS (Murashige and Skoog) medium. (B) Displays of WT and mutant seeds 7 days after being planted in ½ MS medium. (C) Germination rates of WT and mutant seeds in 10 days in ½ MS medium. Asterisks indicate the statistical significance of differences in germination rates of mutant lines compared to the WT on the same day. *p < 0.05; **p < 0.01.

The germination rate measurement experiment results, presented in Figure 5, show images of Petri dishes taken on the fifth and seventh days post‐seeding (Figure 5A,B). Analysis indicates complete germination for both WT and mutant plant seeds. Notably, mutant plant seeds exhibited delayed germination compared to WT seeds. While WT seeds germinated 100% on the eighth day after placing in the medium, mutant seeds reached this rate on the 10th day and later (Figure 5C).

### 3.4 Mutation in *SlHP2* and *SlHP3* genes leads to reduction in both stomata number and aperture

**FIGURE 6:**
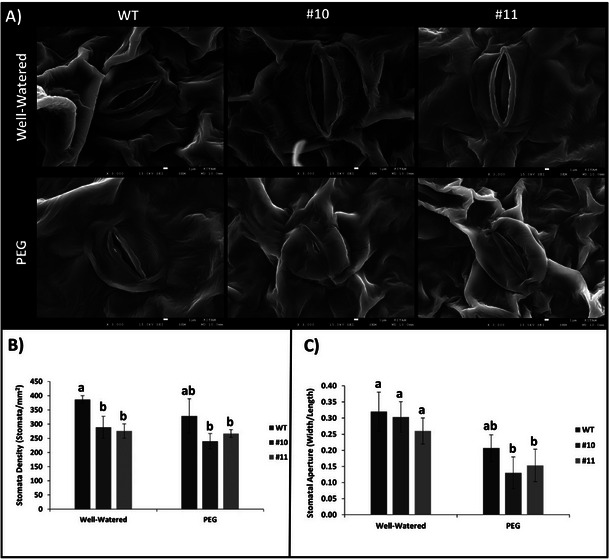
The number of stomata and the stomata aperture in mutant and wild‐type (WT) plants. (A) The electron microscope image of the abaxial surface of the leaf. (B) The average number of stomata in 1 mm^2^ area. (C) The average stomatal aperture of *slhp2,3‐10*, *slhp2,3‐11*, and WT plants. Black lines on B and C show SE ± values for three biological replicates. Different letters above the bars indicate statistically significant differences between groups (*p* < 0.05), as determined by ANOVA followed by Tukey's HSD test. Groups sharing the same letter are not significantly different.

Stomatal morphology is crucial for plant adaptation to drought stress. We examined leaf samples post‐stress using an electron microscope, summarized in Figure 6. Figure 6A displays an electron microscope image of plant leaf abaxial surfaces, while Figure 6B quantifies observed stomata. Remarkably, mutant plants exhibited significant reduced stomatal density compared to WT plants (Figure 6B). While an average of 387 stomata/mm^2^ was observed in the abaxial parts of WT plant leaves under well‐watered conditions, this number was 329 stomata/mm^2^ in PEG‐treated plants. In mutant plant leaves, an average stomatal density of 280 stomata/mm^2^ was observed under well‐watered conditions and 250 stomata/mm^2^ under PEG conditions. However, the significant differences were observed only between the stomatal densities under well‐watered conditions (Figure 6B).

Further analysis, depicted in Figure 6C, revealed differences in stomatal aperture. This emphasizes the pivotal role of *SlHP2* and *SlHP3* signaling in regulating stomatal density and closure during PEG stress. Regarding the stomatal aperture (width/length), it was calculated as 0.32 for WT and *slhp2,3‐10* under well‐watered conditions, while the stomatal aperture of *slhp2,3‐11* was calculated as 0.26. Under PEG conditions, the ratios were 0.21, 0.13, and 0.15 for WT, *slhp2,3‐10*, and *slhp2,3‐11* (Figure 6C).

**FIGURE 7:**
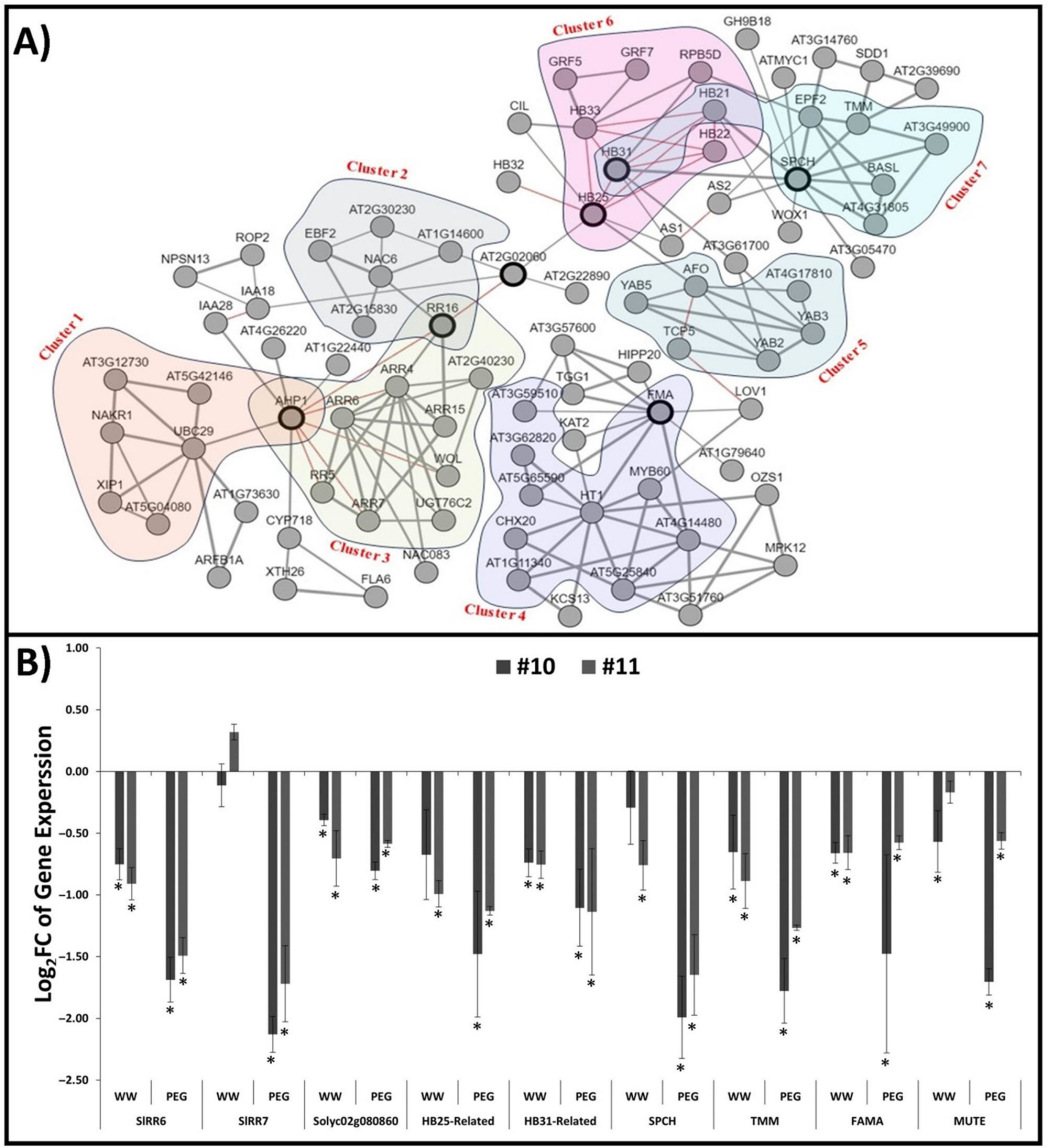
Signal transduction to stomatal differentiation genes through Zinc Finger Homeodomain Proteins and cytokinin signaling regulators. (A) The network map was employed the co‐expression network visualization tool Atted II, developed by Obayashi et al. in 2007. Red lines represent protein–protein interactions, while black lines represent co‐expression. (B) The representation of the expression of homologous genes in mutant lines compared to wild‐type (WT) plants in Log2FC format. Black lines on B represent SE ± values for three biological replicates. Asterisks indicate the significance of gene expression in mutant lines compared to the WT (*p < 0.01).

To investigate the potential roles of cytokinin signaling regulators in stomatal development, we employed both PPI and co‐expression approaches, incorporating the stoma differentiation core gene set (*SPCH*, *FAMA*, *MUTE*, and *TMM*) alongside cytokinin regulators (Figure 7). This methodology enabled the construction of a comprehensive co‐expression signaling network (Figure 7A). Notably, zinc finger homeodomain proteins, namely, HB25, HB31, and *Solyc02g080860*, emerged as co‐expressed entities within this network (Figure 7A). Subsequently, leveraging this co‐expression network, we identified homologs of the Arabidopsis genes in *S*. *lycopersicum* through BLAST analysis. We further evaluated their expression levels using quantitative real‐time PCR (qRT‐PCR) (Figure 7B).

Our findings revealed distinct expression patterns under different conditions. Specifically, the *SPCH* gene exhibited downregulation under normal conditions, while under stress conditions, its expression was upregulated in mutant lines. Conversely, the *MUTE* gene showed upregulation under normal conditions but downregulation under stress conditions. The expression of *Solyc02g080860*, *FAMA*, and *TMM* genes were consistently downregulated under both control and stress conditions. Similarly, genes related to *HB31*, *HB25*, *RR6*, and *RR7* displayed analogous expression responses (Figure 7B). This result suggests that the decreased expression of cytokinin signaling and stomatal formation and differentiation marker regulators may be associated with the reduced stomatal density observed in mutant plants (Figure 6B).

## 4 DISCUSSION

Signal transduction and response in plants, specifically cytokinin signaling, are conceptualized as TCSs that involve the integration of three key signaling elements: receptor histidine kinases, histidine phosphotransfer proteins (HPs), and cytokinin RRs. In the context of this pathway, utilizing *Arabidopsis* as a model organism, histidine phosphotransfer proteins (AHPs) serve as immediate downstream components of AHK receptors. AHPs play a crucial role in facilitating direct phosphotransfer events, thereby orchestrating the activation of type‐B response regulators (type B‐ARRs). This intricate cascade of events contributes to the amplification of cellular responses and the modulation of gene expression patterns in response to cytokinin signals in plants (Schaller et al., 2008; To & Kieber, 2008). In this feedforward mechanism, type‐B ARRs receive a phosphoryl group from AHPs, thereby enabling them to activate the expression of downstream *type‐A ARR* genes (Mason et al., 2005). Furthermore, the direct binding activity of type‐B ARRs to their target sites has been experimentally confirmed in planta following the application of cytokininKs (Zubo et al., 2017). Consistent with their important role in the cytokinin response (Hill et al., 2013) and plant development (F. Zhang et al., 2017), B‐type ARRs are involved in the abiotic stress response and negatively affect plant response to drought.

The elements of the TCS in cytokinin signaling are presently drawing considerable attention in plant research. This increased interest stems from their involvement in diverse abiotic stress responses, influencing hormonal cross‐regulation mechanisms (Kurepa et al., 2014; Xie et al., 2018), lateral root initiation (Moreira et al., 2013), and antioxidant defense (Wen et al., 2015). These initiatives seek to reveal novel TCS elements that play roles in plant growth and development, further exploring their potential contributions to drought stress responses.

In this study, two different genes (*Solyc01g080540* and *Solyc06g084410*) encoding histidine‐containing phosphotransfer in tomato were mutated using CRISPR/Cas9 system and then PEG‐induced stress responses were evaluated by measuring various parameters in the WT and mutant plants. Figure 1 shows the mutations in the plant lines used for the stress experiment. In mutant line 10, an insertion in the *Solyc01g080540* gene and a deletion in the *Solyc06g084410* gene were determined by Sanger sequencing using the ICE‐synthego CRISPR analysis tool. Using the same analysis program, it was determined that there was an insertion in the *Solyc01g080540* gene and two insertions in the *Solyc06g084410* gene in mutant line 11. Since these mutations cause frame shifts, line 10 and line 11 are selected for further experimentation. This study highlights the potential and limitations of CRISPR/Cas9‐mediated gene editing in enhancing stress tolerance in plants. Although CRISPR/Cas9 offers precise targeting, off‐target effects remain a concern, especially for genes linked to complex stress responses. To address this, two different gRNA design tools—such as Benchling and CRISPR‐P, which are widely used and cited in similar research—were employed to minimize off‐target probabilities as much as possible. While these advanced tools enhance reliability, cost remains a significant factor in functional studies, and complete elimination of off‐target risks ultimately requires whole‐genome sequencing (Cameron et al., 2017; Guo et al., 2023; Yan et al., 2020). Additionally, PEG‐induced stress, though useful in controlled experiments, may not fully replicate natural drought conditions. Future advancements could involve multiplex gene editing to target multiple stress‐related genes simultaneously, enhancing resilience. However, regulatory challenges for CRISPR‐modified plants in commercial agriculture persist. Integrating CRISPR with transcriptomics and metabolomics may offer deeper insights into stress response mechanisms, ultimately leading to more effective crop improvement strategies (Rai et al., 2023; Zafar et al., 2020).

Figure 2A shows the morphology of the plants after 7 days of PEG treatment. Plants were allowed to develop to the five‐leaf stage for the onset of stress. Height measurements were taken from the plants that reached the five‐leaf stage and their height at the beginning of the stress is presented (Figure 2B). Although the difference in plant height at the five‐leaf stage was not significant, the mean height of WT plants was slightly higher than the mean height of mutant plants. Although there was no significant difference in plant height after stress, WT plants in well‐watered conditions were slightly taller than mutant plants in the same conditions. There are studies showing that PEG treatment inhibits tomato growth (Wickramasinghe & Seran, 2019). Figure 2B shows that WT and mutant plant heights were negatively affected by PEG conditions. Proline accumulation is known to happen in situations when there is a water shortage (Hare et al., 1998), salt (Munns, 2005; Rhodes et al., 2002), low temperature (Naidu et al., 1991), exposure to heavy metals (Bassi & Sharma, 1993; Sharma & Dietz, 2006), UV radiation, and so on. In addition to serving as an osmolyte for osmotic adjustment, proline also scavenges free radicals, buffers cellular redox potential during stress, and stabilizes subcellular structures including membranes and proteins (Ashraf & Foolad, 2007). In our study, no significant difference was observed between the mutant lines and WT in terms of proline content in both conditions. However, the amount of proline in plant leaves increased significantly under stress conditions as expected (Figure 2C). NCED is the primary enzyme in the plant abscisic acid (ABA) biosynthesis pathway, according to biochemical and genetic research (M. Zhang et al., 2009). The *RD22* gene has been shown to be involved in the response to dehydration and is stimulated by ABA (Yamaguchi‐Shinozaki & Shinozaki, 1993). Figure 2D shows the expression of *NCED* and *RD22* encoding genes under PEG treatment compared to plants under well‐watered conditions. Although the expression of *NCED* and *RD22* genes increased in all plants, including WT, the increase in gene expression in mutant plants was significantly higher compared to WT. Looking at the gene expressions, we can conclude that either the mutant plants were more sensitive to PEG stress and as a result overexpressed these genes, or they were able to perceive PEG stress earlier and so adjust to these conditions more quickly. As a result of peroxidation of polyunsaturated fatty acids, which are part of the biomembrane, reactive by‐products can be formed and one of these products is MDA (Yamauchi et al., 2008). Stress conditions such as drought are known to increase MDA content in tomato leaves (Jia et al., 2023). To understand how mutant plants are affected at the cellular level under PEG conditions, MDA content determination was performed. The MDA results showed that all plant lines had MDA levels of about 5 µmole/g FW when they were well‐watered. These amounts increased to approximately 8 µmole/g FW in mutant lines and 10 µmole/g FW in WT plants under PEG conditions; the amount in WT plants had been found to be significantly higher than in mutant plants (Figure 2E). According to MDA levels, the stress effect of PEG on WT plants was greater than that on mutant plants. A well‐established technique for determining membrane permeability in connection to environmental stressors, growth and development, and genotypic variation is to measure solute leakage from plant tissue (Whitlow et al., 1992). To test the accuracy of the MDA results, we performed electrolyte leakage measurement, another test of membrane permeability. Figure 2F shows the percentage representation of electrolyte leakage. The amount of electrolyte leakage in both WT and mutant lines was around 10% under well‐watered conditions and not statistically different from each other, while it was around 32% in WT plants and 26% in mutant plants under PEG conditions, and these amounts were calculated to be statistically different from each other.

Plants were imaged from above to better understand the effects of PEG treatment on WT and mutant plant growth (Figure 3A). WT plant leaves in well‐watered conditions lost less water at the end of the 4‐h water loss experiment (Figure 3B). On the other hand, when the same experiment was performed on plant leaves taken under PEG conditions, the opposite situation emerged and at the end of 4 h, mutant plant leaves lost 8.5%–10% of their weight, while WT plant leaves lost 11% of their weight (Figure 3C). This showed that the mutant plants were actually better adapted to PEG conditions. Moreover, in Figure 3E, it was observed that the relative water content in mutant plant leaves was higher than that in WT plant leaves. A leaf's hydration condition compared to its maximum water retaining capacity at full turgidity is measured by its relative water content (Smart & Bingham, 1974). Relative water content in tomato leaves decreases under drought stress (Zhou et al., 2017). This means that the mutant plants had a higher water content at the end of the stress. Water shortage reduces the leaf area and chlorophyll content in tomato plants, as it does for other plants (Sivakumar et al., 2016; Suminar et al., 2022). Previous studies have suggested a role for leaf shape and size in plant adaptation to stress conditions (Farris, 1984; Schurr et al., 2000). In our study, PEG treatment caused a decrease in total leaf area of both WT and mutant plants (Figure 2D). When leaf areas were compared under PEG conditions, a significant difference was observed between the mutant plants and WT, while under well‐watered conditions, there was also a significant difference between WT and mutant plants. In other words, the difference in PEG conditions may be due to the fact that the mutant plants had less leaf area at the onset of stress. It has been previously emphasized that PEG application has a negative effect on chlorophyll content (Suminar et al., 2022). In our study, PEG application caused a decrease in the amount of chlorophyll in WT plants and this decrease was statistically significant. However, in mutant plants, even though less chlorophyll content was measured in well‐watered conditions compared to WT, no significant difference was observed under PEG conditions compared to WT. More interestingly, when SPAD measurements of the mutant lines under well‐watered and PEG conditions were compared, no statistically significant difference was found between them (Figure 3F). In a study in *A*. *thaliana*, *ahp* mutant plants were shown to have higher chlorophyll index compared to WT under drought conditions (Ha et al., 2022). Our study agrees with these results to a certain extent, because the mutant plants seemed to be less affected by PEG conditions and did not show a significant decrease in chlorophyll content. To ensure that cytokinin signaling was reduced in mutant plants, the expression of genes encoding proteins responsible for some cytokinin signaling was examined (Figure 3G). The expression of cytokinin signaling genes indicates that cytokinin signaling is reduced. For example, the genes *RR5* and *RR1*, which are *type‐A RRs*, are stimulated by activation of *type‐B RRs* by HPs involved in phosphate transfer. The expression of *RR1* and *RR5* encoding genes in the mutants was significantly reduced compared to their expression in WT. In this case, it was concluded that cytokinin signaling is reduced. The expression of other HP coding genes in tomato was also investigated and it was observed that there was a significant decrease in the gene expression of *HP1*, but surprisingly, there was a significant increase in the gene expression of *HP4*.

The effects of PEG application on root development may differ between species and even between genotypes within species (Robin et al., 2021; Wickramasinghe & Seran, 2019). Because of these conflicting effects, in our study, we compared root development in mutant lines with that in WT plants (Figure 4). Although PEG treatment had no significant effect on the main root length or lateral root number of WT plants, the situation was different in WT plants and an increase was observed in both mutant lines (Figure 4B,C). Suppression of *OsHP* coding genes in rice resulted in increased lateral root development (Sun et al., 2014). Furthermore, cytokinin signaling has been shown to have a negative effect on root development by inducing the expression of *AUX/IAA* and *SHY2/IAA3* genes, which are negative regulators of auxin signaling, and by repressing the expression of *AUX1* gene, a positive regulator of auxin transport (Moubayidin et al., 2009; Schaller et al., 2015).

ABA is the main hormone regulating seed dormancy during seed development in the mother plant (Sano & Marion‐Poll, 2021). During seed development, ABA positively regulates reserve accumulation and inhibits embryo formation, whereas ABA produced by zygotic tissues only at late maturation stages has a dominant role in the induction of dormancy (Seo et al., 2016). ABA and cytokinin regulate many developmental and response stages through their opposing relationship (Huang et al., 2018). A characteristic of mature dry seeds is that it allows them to accumulate mRNAs during seed maturation and use them as templates to synthesize proteins during germination. Some of these stored mRNAs are also called long‐lived mRNAs, as they remain translatable into proteins even after seeds have been exposed to prolonged stressful conditions. Mature seeds can germinate even in the presence of transcriptional inhibitors, and this ability is acquired in mid‐seed development. The type of mRNA accumulated in seeds is influenced by the plant hormone ABA and environmental factors (Galland & Rajjou, 2015; Sajeev et al., 2019). In our study, a significant increase in the expression of genes in the ABA pathway was observed in mutant plants with deficiency in cytokinin signaling. When the germination rate was analyzed, it was observed that slhp mutant lines germinated late compared to WT (Figure 5C).

Plants adjust transpiration rate through various physiological and morphological mechanisms to adapt to changing environmental conditions (Osakabe et al., 2014; Yordanov et al., 2000). Stomata movement is controlled by the circadian rhythm and conditions such as temperature, humidity, water restriction, carbon dioxide, or ABA influence the control of these movements (H. G. Jones, 1998). The stomata of *slhp2,3* mutants and WT plants were examined and the stomata of mutant plants were significantly closed under PEG‐treated conditions (Figure 6C). Different cytokinin derivatives are known to cause stomatal opening (Blackman & Davies, 1983). Therefore, plants with reduced cytokinin signaling might be expected to have more easily closed stomatal structures. However, not only stomatal aperture but also stomatal density is important in the response to water deficit. In a study, it was observed that tomato plants with reduced stomatal density had higher RWC content (Farber et al., 2016). In *Oryza sativa*, the manipulation of stomatal density has been shown to directly affect water use efficiency (WUE) and drought tolerance. For example, research has demonstrated that reduced stomatal density can enhance drought tolerance by minimizing water loss through transpiration while maintaining photosynthesis efficiency. Overexpressing genes like *EPF1* in rice has led to reduced stomatal density and improved WUE under water‐limited conditions (Caine et al., 2019; Phunthong et al., 2024). Similarly, CRISPR/Cas9‐induced mutations in genes such as stomagen and *EPFL10* resulted in a significant reduction of stomatal density, which helped rice plants adapt better to drought stress (Phunthong et al., 2024). In Arabidopsis, wheat, and barley, studies have also shown that stomatal density plays a critical role in regulating plant responses to water scarcity. The *EPF* gene family and other regulatory mechanisms influence stomatal development and water conservation under drought stress (Dunn et al., 2019; Hughes et al., 2017; Jangra et al., 2021). These findings are supported by analogous results in tomato, where stomatal density adjustments have been linked to enhanced drought tolerance and improved WUE.

Figure 7 illustrates a signaling network in Arabidopsis, mapping the transduction of signals to stomatal differentiation genes via AtAHP1 protein, cytokinin regulators, and zinc finger homeodomain proteins. Our findings revealed that only *AtAHP1*—a homolog of *SlHP1* and *SlHP2*—acts as a regulator within this network. To investigate the role of cytokinin signaling in stomatal formation and differentiation, *SlHP1* and *SlHP2* key regulator genes simultaneously mutated in tomato. *slhp2,3* plants formed fewer stomata compared to WT (Figure 6B). If SPCH is absent, the epidermis lacks stomata, and while SPCH protein levels are high, stomatal lineage cells proliferate excessively in *Arabidopsis* (Lampard et al., 2008; MacAlister et al., 2007). The well‐known bHLH transcription factor *FAMA* regulates the terminal differentiation of guard cells master regulator. The differentiation of cells that differentiate to form stomatal structures (mother meristemoid cells, meristemoid, and guard mother cells) is controlled by the dimerization of the bHLH transcription factors (*SPCH*, *MUTE*, and *FAMA*) with *SCRM/ICE1* and *SCRM2* (Lau & Bergmann, 2012; Pillitteri & Torii, 2012). *TMM*, a receptor‐like protein encoding gene, has been shown to positively affect the differentiation process of meristemoid cells into guard mother cells (Bhave et al., 2009). A gene network responsible for stomatal formation and cytokinin signaling was constructed using the AttedII database (Figure 7A). Based on this network, we analyzed the expression of genes involved in stomatal formation, cytokinin signaling, and those serving as intermediaries between these processes (Figure 7B). One of the genes examined through qRT‐PCR was *Solyc02g080860* (the homolog of *AT2G02060*), identified as a crucial link between cytokinin signaling and stomatal formation. While the gene is noted in the literature as part of the Homeodomain‐like superfamily, no further functional studies have explored its specific role. Additionally, two other genes, *SlHB25* and *SlHB31*—homologs of *ZFHD2* (zinc‐finger homeodomain 2) and *ZHD4* in Arabidopsis, respectively—were also found to be essential for connecting cytokinin signaling with stomatal formation. Research on *ZHD2* and *ZHD4* in Arabidopsis has shown their key roles in the transition from meristem to flower organ development and in female gametophyte formation (Pagnussat et al., 2007; Torti et al., 2012). Notably, almost all genes within this network were expressed at lower levels under all conditions compared to WT.

This work aimed to examine the roles played by the *SlHP2* and *SlHP3* genes in tomatoes by CRISPR/Cas9 technology under osmotic stress conditions. Our findings indicate that the expression of these genes contributes to the plant's reduced resilience by limiting root development—a crucial morphological adaptation for water uptake under drought conditions. Additionally, *SlHP2* and *SlHP3* appear to suppress ABA signaling, a critical pathway for drought response. While reduced stomatal density negatively impacts growth and development, it supports WUE by minimizing transpirational water loss from leaves. Overall, this research enhances our understanding of gene function in stress responses in tomatoes and provides a foundation for future genetic improvements targeting these pathways.

## AUTHOR CONTRIBUTIONS

**Abdullah Aydin**: Conceptualization; data curation; methodology; writing—original draft; writing—review and editing. **Bayram Ali Yerlikaya**: Conceptualization; data curation; methodology; writing—original draft. **Seher Yerlikaya**: Conceptualization; data curation; methodology; writing—original draft. **Nisa Nur Yilmaz**: Conceptualization; methodology; writing—original draft. **Musa Kavas**: Conceptualization; data curation; methodology; project administration; resources; writing—original draft; writing—review and editing.

## CONFLICT OF INTEREST STATEMENT

The authors declare no conflicts of interest.

## Supporting information




**Supplementary Figure 1**. The prediction of gRNA secondary structures. gRNA 3 (A) and gRNA 25 (B) targeting *SlHP2*. gRNA 29 (C) and gRNA 78 (D) targeting *SlHP3*. The scale bar represents base‐pair probabilities. Targeting sites of gRNAs (E).

**Supplementary Figure 2**. The constructed *SlHP2,3* pHSE401 vector (A). The agarose gel image of the PCR conducted using *HPTII* gene‐specific primers (B).

**Supplementary Figure 3**. Mutation analysis results with ICE‐Synthego.

**Table S1**. Primer used for clonning.

**Table S2**. Primers used for mutation and T‐DNA insertion check.

**Table S3**. Ortologous Genes.

**Table S4**. Primers used for qPCR.

## Data Availability

All data generated or analyzed during this study are included in this published article (and its Supporting Information files). The materials used in our study are available under an MTA from the corresponding author upon reasonable request.
