# Supplementary figures and images for: CRISPR‐mediated mutation of cytokinin signaling genes (SlHP2 and SlHP3) in tomato: Morphological, physiological, and molecular characterization
Source: Plant Genome. 2025 Jan 8;18(1):e20542. doi: 10.1002/tpg2.20542 (PMC11711121; doi:10.1002/tpg2.20542)

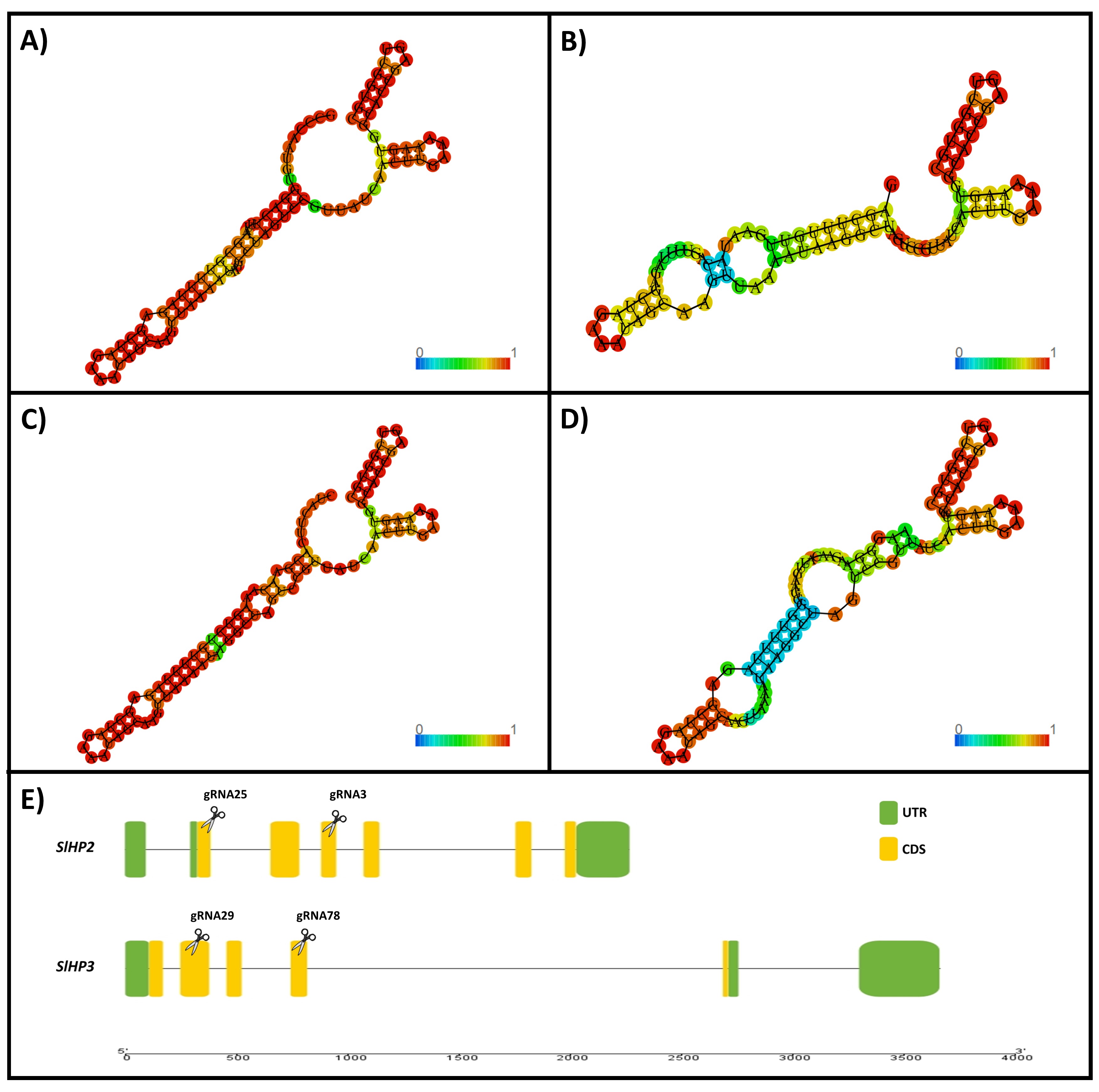

Supplement: Supplementary file 1 — Supplementary Figure 1. The prediction of gRNA secondary structures. gRNA 3 (A) and gRNA 25 (B) targeting SlHP2. gRNA 29 (C) and gRNA 78 (D) targeting SlHP3. The scale bar represents base‐pair probabilities. Targeting sites of gRNAs (E). [file TPG2-18-e20542-s001.jpg]

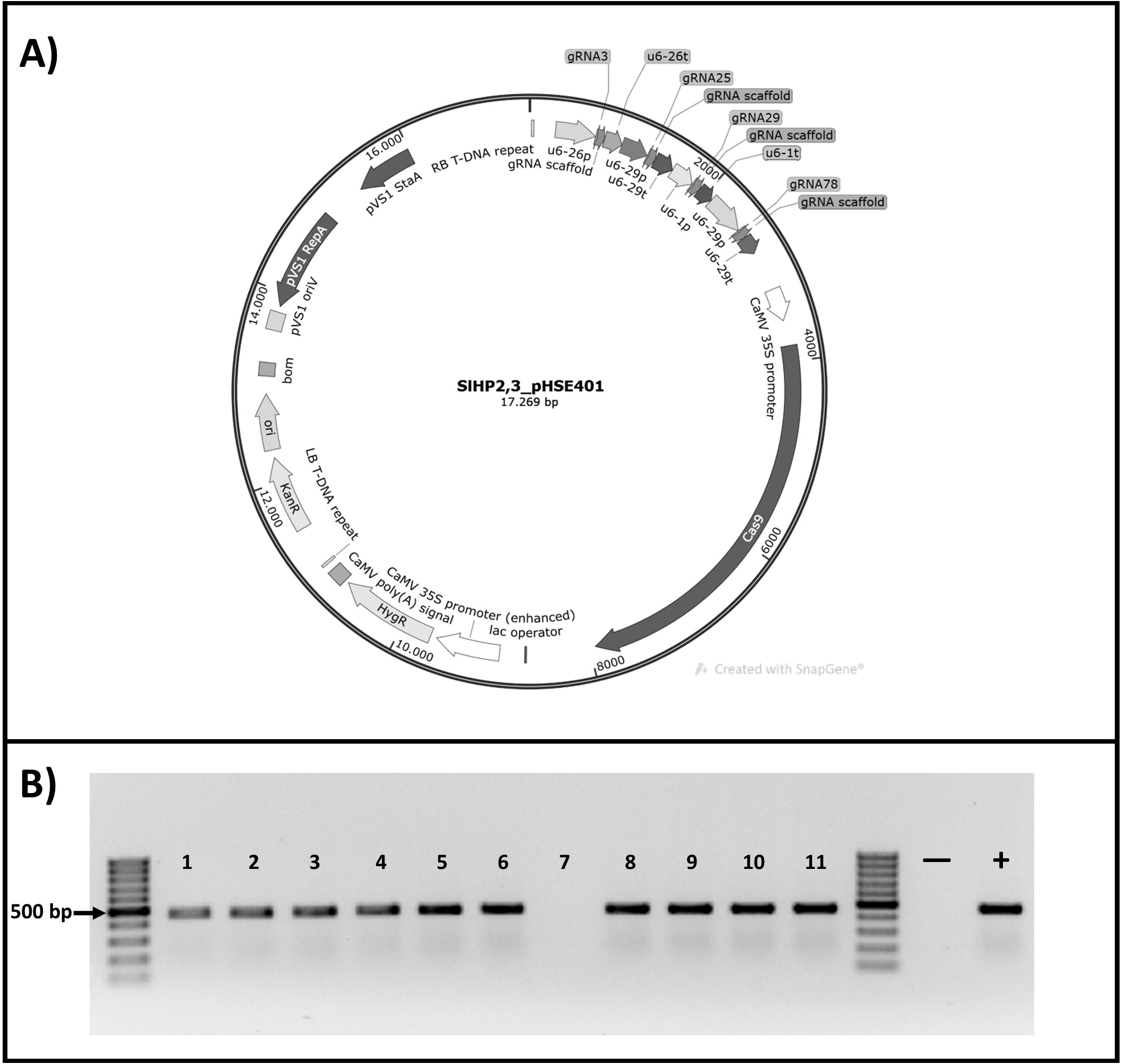

Supplement: Supplementary file 2 — Supplementary Figure 2. The constructed SlHP2,3 pHSE401 vector (A). The agarose gel image of the PCR conducted using HPTII gene‐specific primers (B). [file TPG2-18-e20542-s003.jpg]

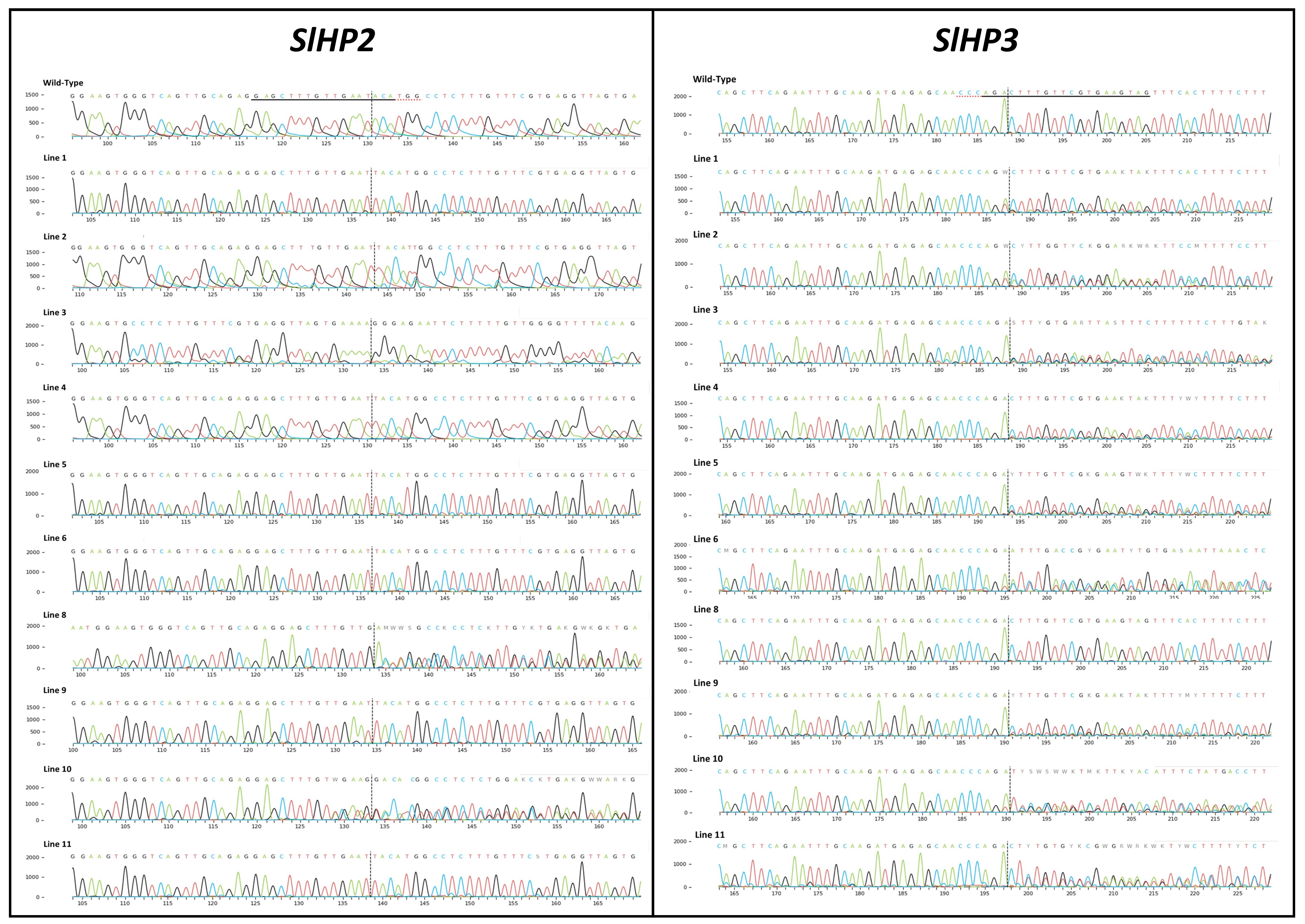

Supplement: Supplementary file 3 — Supplementary Figure 3. Mutation analysis results with ICE‐Synthego. [file TPG2-18-e20542-s004.jpg]
